# Alcoholic Hepatitis Markedly Decreases the Capacity for Urea Synthesis

**DOI:** 10.1371/journal.pone.0158388

**Published:** 2016-07-05

**Authors:** Emilie Glavind, Niels Kristian Aagaard, Henning Grønbæk, Holger Jon Møller, Nikolaj Worm Orntoft, Hendrik Vilstrup, Karen Louise Thomsen

**Affiliations:** 1 Department of Hepatology and Gastroenterology, Aarhus University Hospital, Aarhus, Denmark; 2 Department of Clinical Biochemistry, Aarhus University Hospital, Aarhus, Denmark; University of Louisville School of Medicine, UNITED STATES

## Abstract

**Background and Aim:**

Data on quantitative metabolic liver functions in the life-threatening disease alcoholic hepatitis are scarce. Urea synthesis is an essential metabolic liver function that plays a key regulatory role in nitrogen homeostasis. The urea synthesis capacity decreases in patients with compromised liver function, whereas it increases in patients with inflammation. Alcoholic hepatitis involves both mechanisms, but how these opposite effects are balanced remains unclear. Our aim was to investigate how alcoholic hepatitis affects the capacity for urea synthesis. We related these findings to another measure of metabolic liver function, the galactose elimination capacity (GEC), as well as to clinical disease severity.

**Methods:**

We included 20 patients with alcoholic hepatitis and 7 healthy controls. The urea synthesis capacity was quantified by the functional hepatic nitrogen clearance (FHNC), i.e., the slope of the linear relationship between the blood α-amino nitrogen concentration and urea nitrogen synthesis rate during alanine infusion. The GEC was determined using blood concentration decay curves after intravenous bolus injection of galactose. Clinical disease severity was assessed by the Glasgow Alcoholic Hepatitis Score and Model for End-Stage Liver Disease (MELD) score.

**Results:**

The FHNC was markedly decreased in the alcoholic hepatitis patients compared with the healthy controls (7.2±4.9 L/h vs. 37.4±6.8 L/h, P<0.01), and the largest decrease was observed in those with severe alcoholic hepatitis (4.9±3.6 L/h vs. 9.9±4.9 L/h, P<0.05). The GEC was less markedly reduced than the FHNC. A negative correlation was detected between the FHNC and MELD score (rho = -0.49, P<0.05).

**Conclusions:**

Alcoholic hepatitis markedly decreases the urea synthesis capacity. This decrease is associated with an increase in clinical disease severity. Thus, the metabolic failure in alcoholic hepatitis prevails such that the liver cannot adequately perform the metabolic up-regulation observed in other stressful states, including extrahepatic inflammation, which may contribute to the patients’ poor prognosis.

## Introduction

Alcoholic hepatitis (AH) is a clinical syndrome of jaundice and liver failure in patients with a florid inflammation of the liver due to long-term excessive alcohol intake [1]. The disease carries a high mortality [1, 2], which, besides infections and kidney failure, may be related to functional metabolic deficits in the inflamed liver. However, data on quantitative metabolic liver functions in AH are scarce.

Ureagenesis, i.e. the conversion of amino-nitrogen (N) to urea-N, is an essential metabolic process that occurs exclusively in the liver, without which life could not be sustained, and it has a key regulatory role in N homeostasis. The capacity for urea synthesis is related to functional liver mass, and it is therefore decreased in patients with cirrhosis or compromised liver function due to other causes [3, 4]. A decrease in the urea synthesis capacity compromises the patient’s ability to eliminate potentially toxic levels of nitrogenous substances, and it will eventually result in an increased risk of hepatic encephalopathy. Conversely, the urea synthesis capacity is accelerated in stressful situations like pain, recent surgery, uncontrolled diabetes, and inflammation located outside of the liver (e.g. active inflammatory bowel disease) [5–9]. Moreover, experimentally induced inflammation has been shown to result in an increase in the urea synthesis capacity in rats [10, 11]. Such an increase may contribute to losses of body N and lean body mass and hence present a treat to the integrity of the organism, which may aggravate the clinical course.

AH involves both compromised liver function and liver inflammation, which are expected to decrease and increase urea synthesis, respectively. However, the manner by which these opposing effects on the urea synthesis capacity are balanced in AH patients has never been investigated, even though this information is important for understanding the pathophysiology and clinical risk profile of these patients. This study aimed to clarify this issue by examining how AH affects the capacity for urea synthesis.

To study the regulation of ureagenesis, we quantified the hepatic capacity for urea synthesis by the functional hepatic nitrogen clearance (FHNC). This is a substrate-standardized measure of urea synthesis calculated as the slope of the linear relationship between the blood α-amino-N (AAN) concentration and urea-N synthesis rate (UNSR) during infusion of alanine, which is administered to control the metabolic flux of amino-N [12]. Thus, a decrease in FHNC reflects a decrease in metabolic liver function for amino-N disposal regardless of changes in the concentration of the substrate (blood AAN) involved in the process. We further investigated the galactose elimination capacity (GEC), which reflects the capacity of the exclusively hepatic galactokinase and is therefore used as another quantitative measure of the metabolic capacity of the liver [13]. Notably, both the blood urea concentration and GEC have been identified as markers of mortality in patients presenting with severe forms of alcoholic liver diseases [14–17]. Finally, we related the FHNC to the patients´ GEC, circulating inflammatory markers, clinical disease severity, and hormonal regulators of FHNC [12, 18–21].

## Materials and Methods

### Persons and study design

Twenty consecutive AH patients admitted to the Department of Hepatology and Gastroenterology at the Aarhus University Hospital, Denmark, were included in the study. The AH diagnosis was established by a recent history of alcohol overuse and a combination of biochemical and clinical findings. The diagnosis was verified by liver biopsy for 13 patients, and the histology findings did not refute the clinical diagnosis in any case. The inclusion criteria were as follows: (i) a history of excessive alcohol ingestion prior to admission; (ii) acute jaundice (serum bilirubin ≥80 μmol/L); (iii) absence of bile duct obstruction or other causes of liver disease; and (iv) age ≥18 years. Patients were excluded if any of the following criteria were present: (i) use of either corticosteroids or pentoxifylline for the preceding 8 weeks; (ii) malignancy; (iii) another inflammatory disease; (iv) ongoing gastrointestinal bleeding; or (v) active infection, including spontaneous bacterial peritonitis.

The patients were screened for bacterial infections using blood and urine cultures, chest radiography and, when appropriate, ascitic fluid culture and determination of the leukocyte count. Clinical and laboratory data were recorded on the day of the FHNC assessment. Clinical disease severity was assessed according to the Glasgow Alcoholic Hepatitis Score (GAHS) (severe AH for GAHS≥9) [14] and the Model for End-Stage Liver Disease (MELD) score [22]. GEC and FHNC assessments were performed on successive days.

Seven age- and sex-matched healthy volunteers with no history of liver or other diseases were included as controls for FHNC measurements and standard biochemical analyses (Table 1).

**Table 1:** Baseline characteristics of the patients with alcoholic hepatitis and healthy controls.

	Alcoholic hepatitis patients	Healthy controls
Gender: F/M	3/17	2/5
Age (years) (mean±SD)	51±6	50±8
Weight (kg) (mean±SD)	85.4±19.0	82.3±16.8
Height (cm) (mean±SD)	176±8	179±6
Body mass index (kg/m^2^) (median (IQR))	26.0 (6.7)	24.0 (6.0)
Ascites (yes/no)	11/9	0/7**
Hepatic encephalopathy (yes/no)	4/16	0/7
Aspartate aminotransferase (U/L) (mean±SD)	120±31	32±15*
Alanine aminotransferase (U/L) (median (IQR))	55 (45)	23 (16)*
Aspartate aminotransferase/alanine aminotransferase ratio (mean±SD)	2.3±0.6	1.2±0.4*
Bilirubin (μmol/L) (mean±SD)	282±155	9±2*
Alkaline phosphatase (U/L) (median (IQR))	213 (162)	56 (14)*
Albumin (g/L) (mean±SD)	21±4	37±1*
International normalized ratio (mean±SD)	1.8±0.6	1.1±0.1*
Coagulation factors II, VII, X (median (IQR))	0.33 (0.41)	0.91 (0.23)*
Platelet count (x10^9^/L) (mean±SD)	180±92	240±59
Hemoglobin (mmol/L) (mean±SD)	6.5±1.2	8.6±0.7*
Sodium (mmol/L) (mean±SD)	130±7	139±2*
Urea (mmol/L) (median (IQR))	3.7 (3.3)	4.5 (2.5)
Creatinine (μmol/L) (median (IQR))	62 (36)	78 (10)
C-reactive protein (mg/L) (mean±SD)	31.4±20.7	0.6±0.0*
White blood cell count (x10^9^/L) (mean±SD)	13.5±5.6	5.0±0.9*
Neutrophils (x10^9^/L)	9.5±4.6	2.6±0.9*
Soluble CD163 (mg/L) (mean±SD)	19.4±6.6	1.6±0.3*
Interleukin-6 (ng/L) (median (IQR))	25.4 (44.4)	NA
Tumor necrosis factor α (ng/L) (median (IQR))	2.8 (1.5)	NA

* P<0.01 compared with alcoholic hepatitis patients.

** P<0.05 compared with alcoholic hepatitis patients.

NA = not applicable; IQR = interquartile range.

This study conformed to the Declaration of Helsinki, and written informed consent was obtained from all persons before participation. The protocol was approved by the Central Denmark Region Committees on Health Research Ethics (Journal No. 1-10-72-55-12) and was registered at ClinicalTrials.gov (NCT01245257).

### Investigations

#### FHNC

The investigation was performed after an overnight fast and lasted for 7 h. Alanine (Ajinomoto Co., Inc., Tokyo, Japan) was infused through a catheter inserted into a dorsal hand vein at a constant rate of 2.0 mmol/kg/h with a volumetric pump for 4 h. The time is presented relative to the start of alanine infusion. Initially (-60 min to 0 min) and during the last 2 h (240 min to 360 min), no infusion was administered. Blood samples were collected from a catheter inserted into an antecubital vein on the opposite arm. The blood AAN and urea-N concentrations were measured at -60 min and then at every hour. The participants were encouraged to drink a minimum of 200 ml tap water per hour or were administered an equivalent volume of saline by infusion to maintain high urine production. The bladder was emptied via voiding at hourly intervals. Ultrasound scanning was performed to look for residual urine at first voiding, and for some patients, it was necessary to insert a catheter to empty the bladder. The hourly blood samples were obtained immediately before the bladder was emptied. Urine was collected quantitatively, and the samples were frozen at -20°C for later determination of the urine urea-N concentrations.

Blood samples collected at -60 min were used for standard biochemical analysis, including determination of the plasma aspartate aminotransferase, alanine aminotransferase, bilirubin, alkaline phosphatase, albumin, coagulation factors II, VII, and X, hemoglobin, sodium, urea, creatinine, C-reactive protein (CRP), neutrophils, ammonium and glucose concentrations (the latter was measured again at 360 min), as well as the platelet count, international normalized ratio (INR), and white blood cell count (WCC). These blood samples were analyzed immediately after collection. Furthermore, blood samples for determination of the plasma interleukin-6 (IL-6), tumor necrosis factor α (TNF-α) and serum soluble CD163 (sCD163) levels were collected at -60 min, and blood samples for determining the plasma glucagon and serum cortisol, insulin and insulin-like growth factor-I (IGF-I) levels were collected at -60 and 360 min. These samples were stored at -80°C until further analysis.

#### GEC

The GEC was determined after an intravenous injection of galactose (480 mg/kg) using blood concentration decay curves corrected for urinary excretion as described by Tygstrup [13] for all patients except for 2 (1 patient who died before the GEC was measured and 1 patient whose data were lost due to technical problems).

### Analyses

The blood AAN concentration was measured by the dinitrofluorobenzene method [23], and the urine and blood urea-N concentrations were measured by the urease-Berthelot method [24]. The plasma glucagon level was measured by radioimmunoassay (EMP Millipore Corporation, Billerica, MA, USA). In addition, the serum cortisol level was assessed with a specific enzyme immunoassay (DRG Diagnostics, Marburg, Germany), the serum insulin level was measured with an insulin DELFIA^®^ assay (PerkinElmer, Waltham, MA, USA), and the total serum IGF-I level was measured after acid-ethanol extraction with a validated in-house sandwich assay as previously described [25] with slight modifications, such as the replacement of a secondary detection antibody with a biotinylated IGF-I antibody (catalog # I-8773, Sigma, St. Louis, MO, USA). Further, the plasma IL-6 and TNF-α levels were determined with highly sensitive IL-6 and TNF-α immunoassays, respectively (R&D Systems, Minneapolis, MN, USA). Finally, the serum sCD163 concentration was measured with an in-house sandwich enzyme-linked immunosorbent assay (ELISA), as previously described in detail [26]. Standard biochemical analysis was performed according to accredited laboratory methods at the Hospital Department of Clinical Biochemistry.

### Calculations

Within each hour of observation, the UNSR (mmol/h) was calculated as the urinary excretion rate (E) corrected for the accumulation (A) in total body water (TBW) and for fractional intestinal loss (L) as follows:

UNSR=(E+A)/(1−L)

where E = (urine flow, L/h) x (urinary urea-N, mmol/l), A = (change in blood urea-N, mmol/(L x h)) x (TBW, L) and L is assumed to be 0.14 [27].

TBW was assessed from the body weight (BW, kg), body height (BH, cm) and age (Y, years) with the following formulas:

TBW=0.3625×BW+0.2239×BH−0.1387×Y−14.47

for men, and

TBW=0.2363×BW+0.1962×BH−0.0272×Y−10.26

for women [28].

The FHNC (L/h) was calculated for each person as the slope of the linear regression analysis of UNSRs on corresponding average blood AAN concentrations [12].

### Statistical analysis

Paired and unpaired Student´s t-tests were performed to examine the differences within and between groups. Logarithmic transformation was applied as appropriate to ensure that the variables were normally distributed. The assumption of normality was checked using quantile-quantile plots (Q-Q plots) and histograms. Data exhibiting skewed distributions despite logarithmic transformation were examined using the nonparametric Wilcoxon signed-rank test or Wilcoxon rank-sum test as appropriate. Fisher´s exact test was used to assess differences in proportions. One-way analysis of variance (ANOVA) was used for more than two groups, and significant results were further analyzed using post hoc tests. The Spearman rank-order correlation coefficient (rho) was used to estimate the associations between variables in the patients with alcoholic hepatitis. Multiple linear regression was used to adjust for potential covariates including gender, age, and body mass index and estimate the adjusted mean difference in FHNC between the healthy controls and alcoholic hepatitis patients. Multiple linear regression was also used to address associations between FHNC and significant variables from the Spearman rank-order correlation coefficient analysis.

Normally distributed data are presented as the mean ± standard deviation (SD), and skewed data are presented as the median and interquartile range. A two-tailed P value of ≤0.05 was considered statistically significant. Statistical analysis was performed using Stata 12.1 software from Stata Corporation (College Station, TX, USA).

## Results

### Patient characteristics

The clinical and laboratory data are provided in Table 1. Eleven patients (55%) had severe AH, with a GAHS≥9. All 13 liver biopsies showed histological features of alcoholic steatohepatitis, and 8 (62%) also showed cirrhosis.

### FHNC

**Fig 1:**
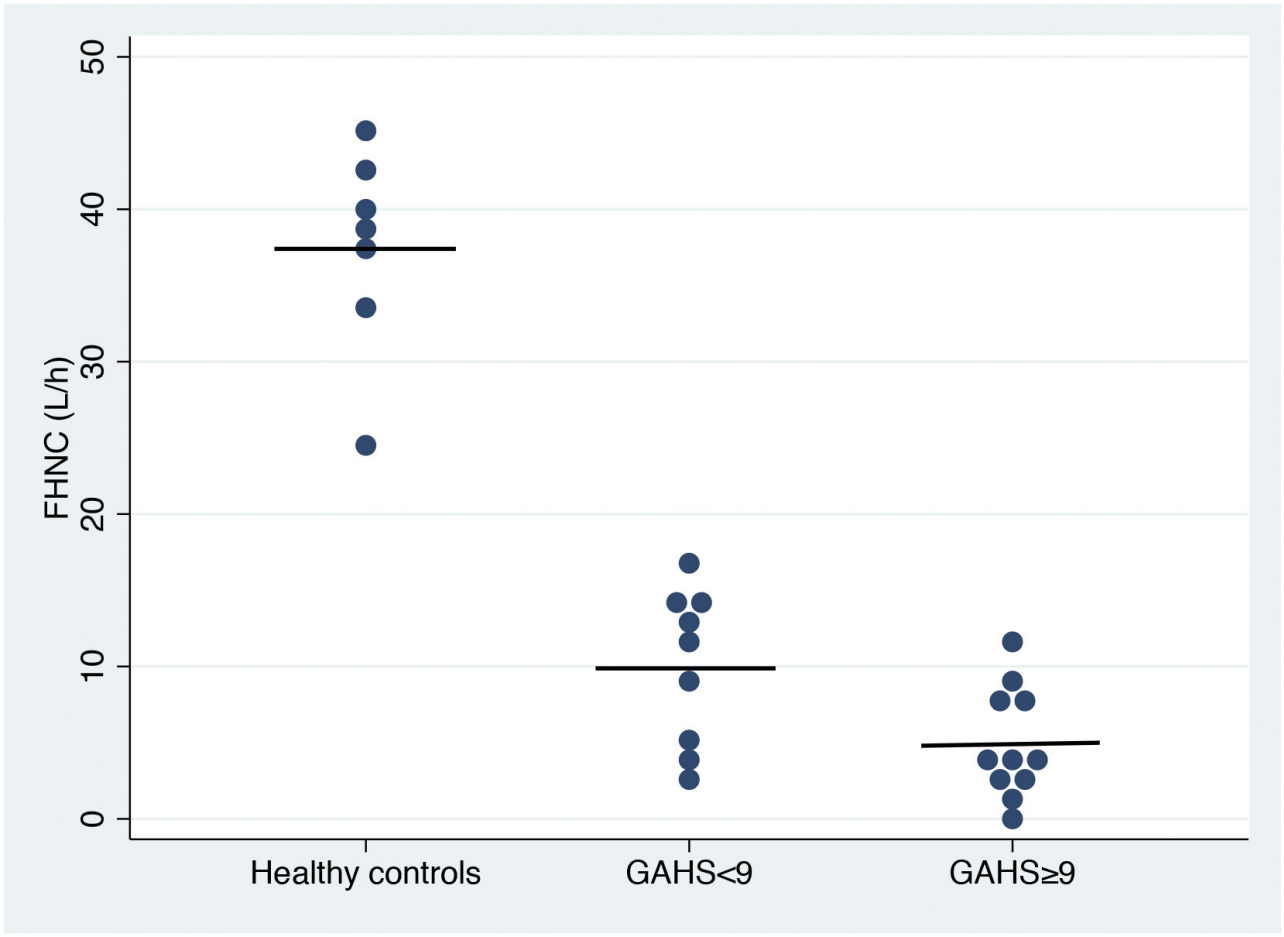
Functional hepatic nitrogen clearance in patients with non-severe and severe alcoholic hepatitis and healthy controls. Functional hepatic nitrogen clearance (FHNC) in patients with non-severe (GAHS<9, N = 9) and severe (GAHS≥9, N = 11) alcoholic hepatitis and healthy controls (N = 7). The solid horizontal lines indicate the mean values. The FHNC was decreased in the AH patients compared with the healthy controls (P<0.01) and the largest decrease was observed in those with severe AH (P<0.05).

The baseline blood AAN concentration was normal in the AH patients (3.3±0.5 mmol/L vs. control 3.3±0.4 mmol/L), and alanine infusion gradually increased it to 10.3±2.1 mmol/L in the AH patients and to 7.3±1.0 mmol/L in the healthy controls (P<0.01). The FHNC was decreased to one fifth in the AH patients compared with the healthy controls (7.2±4.9 L/h vs. 37.4±6.8 L/h; P<0.01) and exhibited the greatest decrease in the patients with severe AH compared with those with non-severe AH (4.9±3.6 L/h vs. 9.9±4.9 L/h; P<0.05) (Fig 1). Results of the multiple linear regression analyses are shown in S1 Table and S2 Table.

### GEC

**Fig 2:**
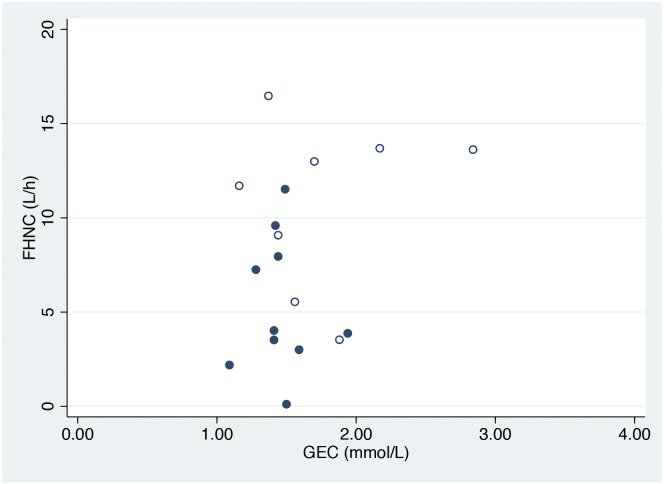
Relationship between the functional hepatic nitrogen clearance and galactose elimination capacity. Relationship between the functional hepatic nitrogen clearance (FHNC) and galactose elimination capacity (GEC) in patients with non-severe (hollow circles) (GAHS<9, N = 8) and severe (circles) (GAHS≥9, N = 10) alcoholic hepatitis. No significant correlation was observed between the FHNC and GEC.

The GEC of the patients was 1.47 (0.29) mmol/min, which was 50% of the expected value, and there was no difference between the patients with severe versus non-severe AH (1.43 (0.09) versus 1.63 (0.62) mmol/min; P>0.05). No correlation was observed between the GEC and FHNC (Fig 2).

### Inflammatory markers

The levels of all inflammatory markers were increased in the AH patients compared with the healthy controls (Table 1). A negative correlation was found between the FHNC and the sCD163 (rho = -0.48; P<0.05), IL-6 (rho = -0.55; P = 0.01) and CRP levels (rho = -0.51; P<0.05). No correlation was detected between the FHNC and WCC, neutrophils, or TNF-α level. Further, no correlation was observed between the GEC and any of the inflammatory markers.

### MELD score and coagulation factors II, VII, and X

**Fig 3:**
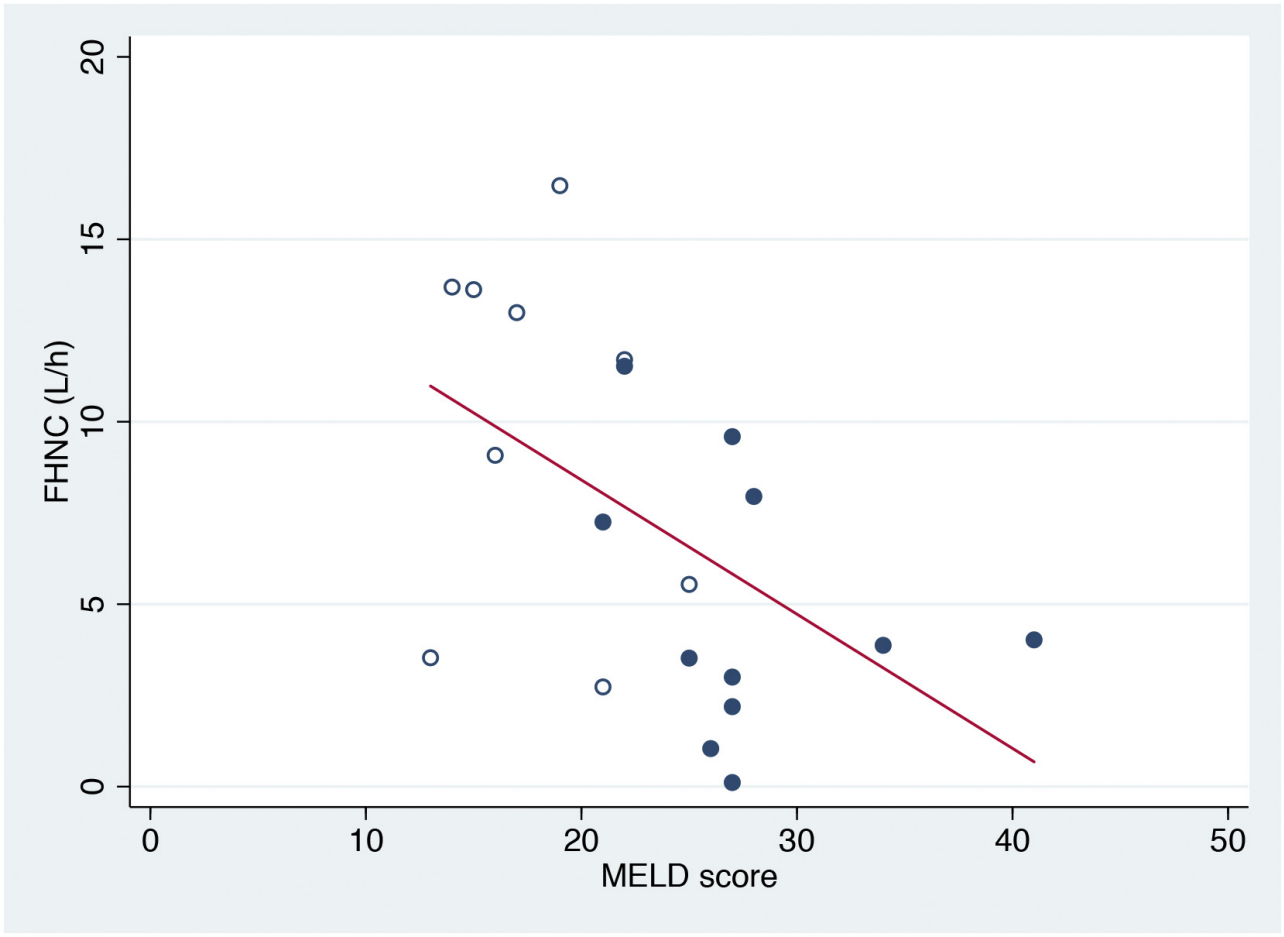
Relationship between the functional hepatic nitrogen clearance and Model for End-Stage Liver Disease score. Relationship between the functional hepatic nitrogen clearance (FHNC) and Model for End-Stage Liver Disease (MELD) score in patients with non-severe (hollow circles) (GAHS<9, N = 9) and severe (circles) (GAHS≥9, N = 11) alcoholic hepatitis. The linear regression line shows the correlation (rho = -0.49; P<0.05).

The MELD score of the patients was 23±7, reflecting advanced liver disease. A negative correlation was found between the FHNC and MELD score (rho = -0.49; P<0.05) (Fig 3). The coagulation factors II, VII, and X were decreased in the patients (P<0.01) and correlated with the FHNC (rho_=_0.44; P = 0.05). No correlation was observed between the GEC and MELD score or coagulation factors II, VII, and X.

### Plasma glucagon, cortisol, IGF-I, insulin and glucose levels

The baseline glucagon concentration was higher and the IGF-I concentration lower in the patients than in the healthy controls (P<0.01) (Table 2). The cortisol, insulin and glucose levels were similar between the two groups. In the AH patients, alanine infusion increased the glucagon (P<0.01) and IGF-I levels (P<0.05) and decreased the cortisol level (P<0.05), whereas the insulin and glucose levels remained unchanged. In the healthy controls, alanine infusion did not affect the glucagon, IGF-I or glucose level, but it decreased the cortisol (P<0.01) and insulin levels (P<0.01).

**Table 2:** Plasma hormone and glucose levels measured before (t = -60 min) and after intravenous alanine infusion (t = 360 min) in patients with alcoholic hepatitis and in healthy controls.

	Time, minutes	Alcoholic hepatitis patients	Healthy controls
Glucagon (ng/L) (median (IQR))	-60	193 (462)	73 (33)***
	360	604 (1223)*	71 (59)***
Cortisol (μg/L) (mean±SD)	-60	109±38	114±24
	360	87±28**	53±23*, ***
Glucose (mmol/L) (median (IQR))	-60	5.7 (1.9)	5.6 (1.0)
	360	6.1 (1.2)	5.1 (0.6)****
Insulin (pmol/L) (median (IQR))	-60	59 (53)	36 (51)
	360	71 (61)	33 (28)*, ***
IGF-I (μg/L) (median (IQR))	-60	28 (39)	138 (38)***
	360	33 (49)**	140 (54)***

*P<0.01 compared with t = -60.

**P<0.05 compared with t = -60.

***P<0.01 compared with alcoholic hepatitis patients.

****P<0.05 compared with alcoholic hepatitis patients.

IGF-I = insulin-like growth factor-I; IQR = interquartile range.

## Discussion

The main finding of this study was that AH markedly decreased the urea synthesis capacity. This decrease was associated with increased clinical disease severity. Thus, the hepatic inflammation down-regulates this essential metabolic liver function instead of up-regulating it, unlike extrahepatic inflammatory states, and hence prevents the liver from adequately taking part in the metabolic up-regulation observed in other stressful conditions.

We established the AH diagnosis using a history of excessive alcohol ingestion and a combination of biochemical and clinical findings, in accordance with the clinical guidelines and clinical trials [14, 29, 30]. Liver biopsy was not a requirement for study inclusion, but it confirmed the clinical AH diagnosis in those for which it was performed. The large prevalence of cirrhosis observed is in agreement with the findings of another recent report [31]. Similarly, the age and gender distributions are similar to those of a previously reported Danish AH population [2]. Notably, our AH patients seemed slightly overweight, which may have contributed to their liver diseases [32]. However, more than half of the patients had ascites; therefore, the use of BMI as a measure of nutritional status should be used with caution in this setting.

It was unclear *a priori* what could be expected from measuring the FHNC in AH. From the perspective of the well-described up-regulation of the FHNC in stressful conditions or in individuals with inflammatory diseases that do not primarily involve the liver, our findings were unexpected, but they were less surprising from a liver failure perspective. In this study, despite the florid inflammatory state, the patients suffered a near-failure of urea synthesis. Alanine infusion resulted in a higher AAN concentration in the AH patients compared with the healthy controls despite the similar baseline AAN levels, which also illustrates the decreased FHNC. The magnitude of the FHNC measured in our healthy controls is similar to previously reported findings [21, 33]. No previous study has investigated the *in vivo* capacity for urea synthesis (i.e., FHNC) in AH, but our findings are in accordance with those of a 42-year-old study demonstrating decreased activity of the urea cycle feeder enzyme, carbamoyl phosphate synthetase, in liver tissues from AH patients [34]. Our findings are also in agreement with those of our previous study reporting a decreased urea synthesis capacity in rats with diet-induced non-alcoholic steatohepatitis (NASH) [35]. A decreased capacity for urea synthesis may lead to hepatic encephalopathy, and 20% of our patients indeed had overt hepatic encephalopathy at the time of the investigation. Unfortunately, we were not able to link these findings to the ammonia concentrations because of issues with analytical interference.

To measure the FHNC, several assumptions, that have been extensively discussed elsewhere [12], must be fulfilled. However, the assumptions pertaining to water movement and urea distribution may be more than commonly critical in our patients with frequent oliguric hepatorenal syndrome. In fact, some of the patients retained water and exhibited a slight increase in body weight during the investigation. However, the error attributed to these effects could not have reached a level that substantially influenced our main results.

The metabolic liver failure was universal in our patients and not limited to urea synthesis, as reflected by the low GEC. However, the metabolic impairments were not concordant. The decrease in the GEC was much less marked than the loss of the urea synthesis capacity, and these two functions were not mutually correlated. The elimination of galactose is not in itself an essential liver function like urea synthesis, and the loss of FHNC probably better reflects the life-threatening metabolic failure that occurs in AH. One explanation for this dissociation may be that urea synthesis involves mitochondrial enzymes, the expression of which we have shown to be markedly reduced by experimental NASH [35]. This finding suggests that the inflammatory fatty liver conditions of both non-alcoholic and alcoholic etiologies share common pathogenic traits, although human NASH data are lacking to date. It is noteworthy that the hepatic synthesis of the acute phase protein CRP seemed to be relatively preserved. This finding may suggest that the hepatic acute phase response is a highly prioritized metabolic liver function in AH patients.

As expected, the patients’ blood inflammatory marker levels were increased, and both TNF-α and IL-6 typically contribute to the inflammation-dependent up-regulation of urea synthesis [11, 36]. However, we found negative correlations between the FHNC and the IL-6 and CRP levels, and as a novel finding, we identified a negative correlation between the FHNC and the level of the macrophage-specific activation marker sCD163 [37]. We have previously demonstrated widespread liver-resident macrophage (Kupffer cell) activation in AH [38], and the present results suggest that this immunogenic mechanism has functional consequences on the inflamed liver, causing it to be metabolically unresponsive to the increased levels of inflammatory markers and mediators. These findings contrast with what occurs in most other inflammatory states, in which the liver is intact and can respond appropriately to regulatory stimuli.

Our results indicated that the degree of failure of urea synthesis was dependent on disease severity. The FHNC was lowest in the patients with AH graded as severe by the GAHS, and it also mirrored the MELD score and levels of external coagulation factors. These results support the notion that the liver inflammation decreases the FHNC by damaging and reducing the liver function. Notably, the FHNC values that we measured in the severe AH patients were as low as those previously reported only in patients with acute liver failure of a non-alcoholic etiology [39]. Such low values have not been reported in even advanced cirrhosis patients [21, 40, 41]. Further, some of our AH patients did not have cirrhosis, and the presence of cirrhosis did not distinguish the particular values of FHNC. Therefore, the functional damage to the liver was likely due to inflammation rather than to cirrhosis.

The low FHNC observed in this study was not due to the insufficient hormonal control of urea synthesis or to an acute alcohol effect. Glucagon is the strongest known up-regulator of urea synthesis [18], and as expected, our patients had hyperglucagonemia in both the basal state and during stimulation with alanine. Further, IGF-I is a strong down-regulator of urea synthesis, and the IGF-I level was low in the patients compared with the healthy controls. In addition, the cortisol, glucose, and insulin levels were within the normal ranges. Alcohol acutely down-regulates the FHNC [33], but our AH patients abstained from alcohol during the days immediately preceding and throughout the investigation.

In conclusion, AH markedly decreases the capacity for urea synthesis, and it does so to a level that has been previously reported only in acute liver failure. In addition, the decrease in urea synthesis capacity is associated with increased clinical disease severity. The metabolic failure in AH results in the inability of the liver to adequately promote the metabolic up-regulation observed in other stressful states involving extrahepatic inflammation. This may compromise the body’s ability to adjust to the homeostatic requirements of the florid inflammation in AH and may contribute to the poor prognosis of these patients.

## Supporting Information

S1 Table Unadjusted and adjusted mean difference in the functional hepatic nitrogen clearance between the healthy controls and alcoholic hepatitis patients using multiple linear regression model. (DOCX)

S2 Table Multiple linear regression model with soluble CD163, interleukin-6, C-reactive protein, MELD score, and coagulation factors II, VII, and X as the explanatory variable for functional hepatic nitrogen clearance in patients with alcoholic hepatitis. (DOCX)
