# Supplementary material for: Alcoholic Hepatitis Markedly Decreases the Capacity for Urea Synthesis
Source: PLoS One. 2016 Jul 5;11(7):e0158388. doi: 10.1371/journal.pone.0158388 (PMC4933397; doi:10.1371/journal.pone.0158388)
Supplement: S1 Table — (DOCX) [file pone.0158388.s001.docx]

**S1 Table. Unadjusted and adjusted mean difference in the functional hepatic nitrogen clearance between the healthy controls and alcoholic hepatitis patients using multiple linear regression model.**

|  | **Mean difference** | **95% CI** | **P-value** |
| --- | --- | --- | --- |
| Unadjusted | 30.2 L/h | 25.3-35.1 L/h | <0.01 |
| Adjusted | 30.9 L/h | 25.9-36.0 L/h | <0.01 |

Unadjusted and adjusted mean difference in the functional hepatic nitrogen clearance between the healthy controls and alcoholic hepatitis patients with 95% confidence intervals (CI) and p-values are presented.

Gender, age, and body mass index (logarithmically transformed) were included in the multiple linear regression model. None of these variables were statistically significant in the model.
