# Supplementary material for: Alcoholic Hepatitis Markedly Decreases the Capacity for Urea Synthesis
Source: PLoS One. 2016 Jul 5;11(7):e0158388. doi: 10.1371/journal.pone.0158388 (PMC4933397; doi:10.1371/journal.pone.0158388)
Supplement: S2 Table — (DOCX) [file pone.0158388.s002.docx]

**S2 Table. Multiple linear regression model with soluble CD163, interleukin-6, C-reactive protein, MELD score, and coagulation factors II, VII, and X as the explanatory variables for functional hepatic nitrogen clearance in patients with alcoholic hepatitis.**

| **Independent variable** | **Regression coefficient** | **P-value** |
| --- | --- | --- |
| Soluble CD163 | -0.21 | 0.22 |
| Interleukin-6* | -0.91 | 0.52 |
| C-reactive protein | -0.07 | 0.27 |
| MELD score | -0.11 | 0.72 |
| Coagulation factors II, VII, and X* | -0.16 | 0.96 |

Regression coefficient and p-value for each independent variable are presented. None of the independent variables were statistically significant in the model. A backward elimination procedure resulted in C-reactive protein being significant in the final model (P<0.05).

* Variable was logarithmically transformed to ensure a normal distribution.

MELD=Model for End-Stage Liver Disease.
